# Evaluation of HIV-1 DNA resistance evolution in highly treatment-experienced and multi-resistant individuals under suppressive antiretroviral therapy: a longitudinal study from the PRESTIGIO Registry

**DOI:** 10.1093/jac/dkaf349

**Published:** 2025-09-24

**Authors:** D Armenia, G Marchegiani, V Spagnuolo, M C Bellocchi, L Galli, T Clemente, L Carioti, R Lolatto, M Ferrara, R Gagliardini, G C Marchetti, C Torti, G De Socio, C Fornabaio, M Zazzi, A Castagna, M M Santoro, PRESTIGIO Study Group, Antonella Castagna, Vincenzo Spagnuolo, Daniele Armenia, Stefano Bonora, Leonardo Calza, Anna Maria Cattelan, Giovanni Cenderello, Adriana Cervo, Laura Comi, Antonio Di Biagio, Emanuele Focà, Roberta Gagliardini, Andrea Giacomelli, Filippo Lagi, Giulia Marchetti, Stefano Rusconi, Francesco Saladini, Maria Santoro, Maurizio Zazzi, Andrea Galli, Daniele Armenia, Francesco Saladini, Maria Santoro, Maurizio Zazzi, Elisabetta Carini, Sabrina Bagaglio, Girolamo Piromalli, Riccardo Lolatto, Nicolò Capra, Marcello Tavio, Alessandra Mataloni Paggi, Silvia Magnani, Manuela Colafigli, Ornella Schioppa, Stefania Zanussi, Valentina Da Ros, Silvia Rossetto, Annalisa Saracino, Flavia Balena, Laura Comi, Daniela Valenti, Pierluigi Viale, Leonardo Calza, Federica Malerba, Silvia Cretella, Riccardo Riccardi, Francesco Castelli, Emanuele Focà, Davide Minisci, Barbara Menzaghi, Maddalena Farinazzo, Chiara Abeli, Bruno Cacopardo, Maurizio Celesia, Michele Salvatore Paternò Raddusa, Carmen Giarratana, Paolo Fusco, Vincenzo Olivadese, Simona Mongiardi, Angelo Pan, Chiara Fornabaio, Paola Brambilla, Alessandro Bartoloni, Filippo Lagi, Paola Corsi, Trevisan Sasha, Gasparro Giuseppe, Cecilia Costa, Alessio Bellucci, Elisa Mariabelli, Teresa Santantonio, Sergio Lo Caputo, Sergio Ferrara, Arianna Narducci, Emanuele Pontali, Marcello Feasi, Antonio Sarà, Matteo Bassetti, Antonio Di Biagio, Sabrina Blanchi, Stefania Piconi, Martina Bottanelli, Silvia Pontiggia, Valsecchi Giada, Stefano Rusconi, Cinzia Roberta Bassoli, Francesco Bassani, Liana Bevilacqua, Antonella Castagna, Vincenzo Spagnuolo, Camilla Muccini, Elisabetta Carini, Sabrina Bagaglio, Riccardo Lolatto, Nicolò Capra, Andrea Galli, Rebecka Papaioannu, Tommaso Clemente, Golnaz Torkjazi, Girolamo Piromalli, Spinello Antinori, Andrea Giacomelli, Tiziana Formenti, Giulia Marchetti, Lidia Gazzola, Fabiana Trionfo Fineo, Massimo Puoti, Cristina Moioli, Federico D’Amico, Simoncini Elena, Sassi Serena, Cristina Mussini, Adriana Cervo, Giulia Nardini, Elio Manzillo, Antonella Gallicchio, Anna Maria Cattelan, Maria Mazzitelli, Antonio Cascio, Marcello Trizzino, Elisa Fronti, Diletta Laccabue, Federica Carli, Roberto Gulminetti, Layla Pagnucco, Mattia Demitri, Alessandra Ferrari, Daniela Francisci, Giuseppe De Socio, Elisabetta Schiaroli, Elisa Garlassi, Romina Corsini, Roberta Gagliardini, Marisa Fusto, Loredana Sarmati, Vincenzo Malagnino, Tiziana Mulas, Mirko Compagno Carlo Torti, Simona Di Giambenedetto, Silvia Lamonica, Pierluigi Salvo, Giovanni Cenderello, Rachele Pincino, Davide Laurenda, Giordano Madeddu, Andrea De Vito, Mario Tumbarello, Massimiliano Fabbiani, Francesca Panza, Ilaria Rancan, Giovanni Di Perri, Stefano Bonora, Micol Ferrara, Andrea Calcagno, Silvia Fantino, Giancarlo Orofino, Guido Calleri, Guastavigna Marta, Stefano Nardi, Marta Fiscon

**Affiliations:** Departimental Faculty, Saint Camillus International University of Health Sciences, Rome, Italy; Department of Experimental Medicine, University of Rome ‘Tor Vergata’, Via Montpellier 1, Rome 00133, Italy; Infectious Diseases Unit, IRCCS San Raffaele Scientific Institute, Milan, Italy; Department of Experimental Medicine, University of Rome ‘Tor Vergata’, Via Montpellier 1, Rome 00133, Italy; Infectious Diseases Unit, IRCCS San Raffaele Scientific Institute, Milan, Italy; Infectious Diseases Unit, IRCCS San Raffaele Scientific Institute, Milan, Italy; Department of Experimental Medicine, University of Rome ‘Tor Vergata’, Via Montpellier 1, Rome 00133, Italy; Infectious Diseases Unit, IRCCS San Raffaele Scientific Institute, Milan, Italy; Department of Medical Sciences, Unit of Infectious Diseases, University of Torino, Torino, Italy; Clinical Infectious Diseases Department, National Institute for Infectious Diseases ‘L. Spallanzani’ IRCCS, Rome, Italy; Clinic of Infectious Diseases, Department of Health Sciences, San Paolo Hospital, ASST Santi Paolo e Carlo, University of Milan, Milan, Italy; Sezione Malattie Infettive, Dipartimento di Sicurezza e Bioetica, Fondazione Policlinico Universitario ‘A. Gemelli’ IRCCS, Rome, Italy; Unit of Infectious Diseases, Santa Maria Hospital, Perugia, Italy; Infectious Diseases Unit, Cremona ASST Hospital, Cremona, Italy; Department of Medical Biotechnology, University of Siena, Siena, Italy; Infectious Diseases Unit, IRCCS San Raffaele Scientific Institute, Milan, Italy; Department of Experimental Medicine, University of Rome ‘Tor Vergata’, Via Montpellier 1, Rome 00133, Italy

## Abstract

**Background:**

This study aimed to clarify whether resistance detected in HIV-1 DNA might evolve in virologically suppressed highly treatment-experienced (HTE) individuals with multidrug resistance (MDR).

**Methods:**

Twenty-three virologically suppressed HTE MDR individuals from the PRESTIGIO Registry with two longitudinal samples available under virological suppression at two different time points (T0−T1) were analysed. HIV-1 DNA levels were quantified using droplet digital PCR, and resistance was assessed through next-generation sequencing (NGS) set at 5%. Mutational load was also evaluated.

**Results:**

At T0, individuals had been virologically suppressed for a median time of 3 years (IQR 3–5) under a salvage regimen, mostly containing dolutegravir (95.7%) and/or darunavir (69.6%). The median HIV-1 DNA level was 2588 copies/10^6^ CD4+ cells at T0 and remained stable at T1 (2322 copies/10^6^ CD4+ cells; *P* = 0.831). Individuals with at least ≥3-class resistance in HIV-1 DNA were 20 (87.0%) at T0 and 18 (78.2%) at T1 (*P* = 0.607). In those receiving NNRTI-sparing treatment (52.2%), the number of NNRTI major resistance mutation (MRM) significantly decreased over time (T0, 2 [1–3]; T1, 0 [0–1]; *P* = 0.027). No significant temporal differences in the number of PI, NRTI and integrase strand transfer inhibitor (INSTI) MRM were found. Specific MRM, such as M184V, decreased over time, particularly in individuals who were receiving a 3TC-/FTC-sparing salvage regimen or with a T0 mutational load of <1000 copies/10^6^ CD4+ cells.

**Conclusions:**

Over a year, HIV-1 DNA MRM generally remained unchanged in suppressed HTE MDR people with HIV (PWH) except for a significant decline in M184V and a reduction of NNRTI resistance in the absence of NNRTI pressure.

## Introduction

Highly treatment-experienced (HTE) people with HIV (PWH) who harbour a multidrug-resistant (MDR) virus represent a particularly fragile population, with an elevated risk of virological failure and disease progression.^1^ These individuals have been typically exposed to multiple antiretroviral therapies (ARTs), often leading to the accumulation of resistance to several drug classes. Despite this, optimized treatment regimens tailored to each individual’s resistance profile can enable HTE individuals to achieve and maintain virological suppression.^2^ In the context of virological control, genotypic resistance testing (GRT) on HIV-1 DNA may help to optimize HIV treatment. Recent findings, including ours, suggested that HIV-1 DNA GRT should be performed through next-generation sequencing (NGS) to allow highly sensitive resistance detection.^3–5^ Advances in NGS technology and increased rates of virological suppression in HTE MDR individuals advise for investigating HIV-1 DNA evolution to assess the possibility of recycling molecules with past resistance. Some studies have shown that mutations such as M184V may decline over time, suggesting that the viral reservoir evolves even in the presence of suppressive ART.^6–8^ The clearance of this mutation seems to be influenced by factors such as CD4 nadir and viral load zenith.^8^ Due to multiple ART failures, the extent of archived HIV resistance might be higher in HTE individuals, but little is known about the evolution of resistance in this population.^9^ Based on these considerations, the present study aimed to investigate whether resistance detected in HIV-1 DNA evolves in virologically suppressed HTE individuals with MDR, providing insights into the viral reservoir dynamics in this fragile population.

## Materials and methods

### Study design

Virologically suppressed (HIV-1 RNA < 50 copies/mL) HTE MDR individuals with at least intermediate resistance documented (based on Stanford HIVdb definition) to at least one drug in each of the four classes of antiretroviral drugs [NRTIs, NNRTIs, PIs and integrase strand transfer inhibitors (INSTIs)] were selected from the PRESTIGIO Registry (https://registroprestigio.org/project).^10^ Inclusion criteria required the availability of two longitudinal peripheral blood mononuclear cell (PBMC) samples, both collected under virological suppression over a time ranging from 9 to 12 months, with the first sample (T0) representing baseline and the second (T1) serving as the follow-up.

### Ethics

PRESTIGIO Registry was approved by Ethics Committees of the coordinating centre (IRCCS San Raffaele Scientific Institute, Milan, Italy; protocol no. 41/int/December 2017) and of all the participating centres.

### HIV-1 DNA quantification and resistance mutation analysis

HIV-1 DNA extraction from PBMCs was performed by using a commercially available kit (High Pure PCR Template Preparation Kit, Roche) and QX200™ Droplet Digital™ PCR System (ddPCR, Bio-Rad, Hercules, CA, USA) was used for total HIV-1 DNA quantification, as previously described.^5^ NGS of HIV-1 protease (PR), reverse transcriptase (RT), integrase (IN) DNA was performed using the MiSeq platform (Illumina Inc.) at both T0 and T1, as previously described.^5^ HIV-1 DNA resistance was assessed with a sensitivity threshold of 5%, and major resistance mutations (MRMs) were identified according to HIVdb version 9.8 (https://hivdb.stanford.edu). Mutational load was calculated by multiplying the mutation frequency detected per contextual HIV-1 DNA load to evaluate changes in mutation burden over time.

### Statistical analyses

Analyses were executed using the SPSS v.26.0 software package for Windows (IBM SPSS Statistics, ver. 26.0). To evaluate differences over time in HIV-1 DNA levels, resistance and mutational load within the same individual, Wilcoxon test and McNemar test for paired samples were used for continuous and categorical variables, respectively.

## Results

### Participants’ characteristics at baseline (T0)

The cohort included 23 HTE MDR individuals with a median age of 55 years (IQR 53–59) at baseline, of whom 73.9% were male. At sampling, the participants had been on ART for a median of 22 years (IQR 21–25), under the current regimen for a median of 37 months (IQR 19–44) and virologically suppressed for a median of 42 months (IQR 31–61). The current salvage regimen was mostly based on dolutegravir (95.7%) and/or darunavir (69.6%); 34.7% of individuals were receiving entry inhibitors [fostemsavir (8.7%), maraviroc (21.7%), enfuvirtide (4.3%)]. Around half of individuals (52.2%) were receiving an NNRTI-sparing regimen (median [IQR] time from class withdrawal: 8 [4–12] years). Similarly, 52.2% of people were receiving a lamivudine-/emtricitabine (FTC/3TC)-sparing regimen (median [IQR] time from drug withdrawal: 8 [4–10] years). All the individuals maintained the same regimen during the follow-up, and the median interval between the two sampling points (T0 and T1) was 12.8 months (IQR 11.8–13.6). Finally, the median (IQR) HIV-1 DNA was 2588 (929–5122) copies per 10^6^ CD4+ cells at T0, and it did not significantly change at T1 (2322 [1521–4138] copies/10^6^ CD4+ cells; *P* = 0.831).

### Dynamics of HIV-1 DNA resistance mutations

Individuals with at least  ≥ 3-class resistance in HIV-1 DNA were 20 (87.0%) at T0 and 18 (78.2%) at T1 (*P* = 0.607). The number of any MRMs and class-specific MRM did not significantly change over time (Table S1, available as Supplementary data at *JAC* Online). Specifically, the median (IQR) number of MRM was 12 (10–16) at T0 and 13 (8–14) at T1 (*P* = 0.384). Considering specific drug classes, the number of NNRTI MRM significantly decreased exclusively in individuals under an NNRTI-sparing treatment (2 [1–3] at T0 and 0 [0–1] at T1; *P* = 0.027). No significant differences from T0 to T1 in the number of PI, NRTI (overall and only thymidine analogue mutations) and INSTI MRM were found (Table S1).

**Figure 1.**
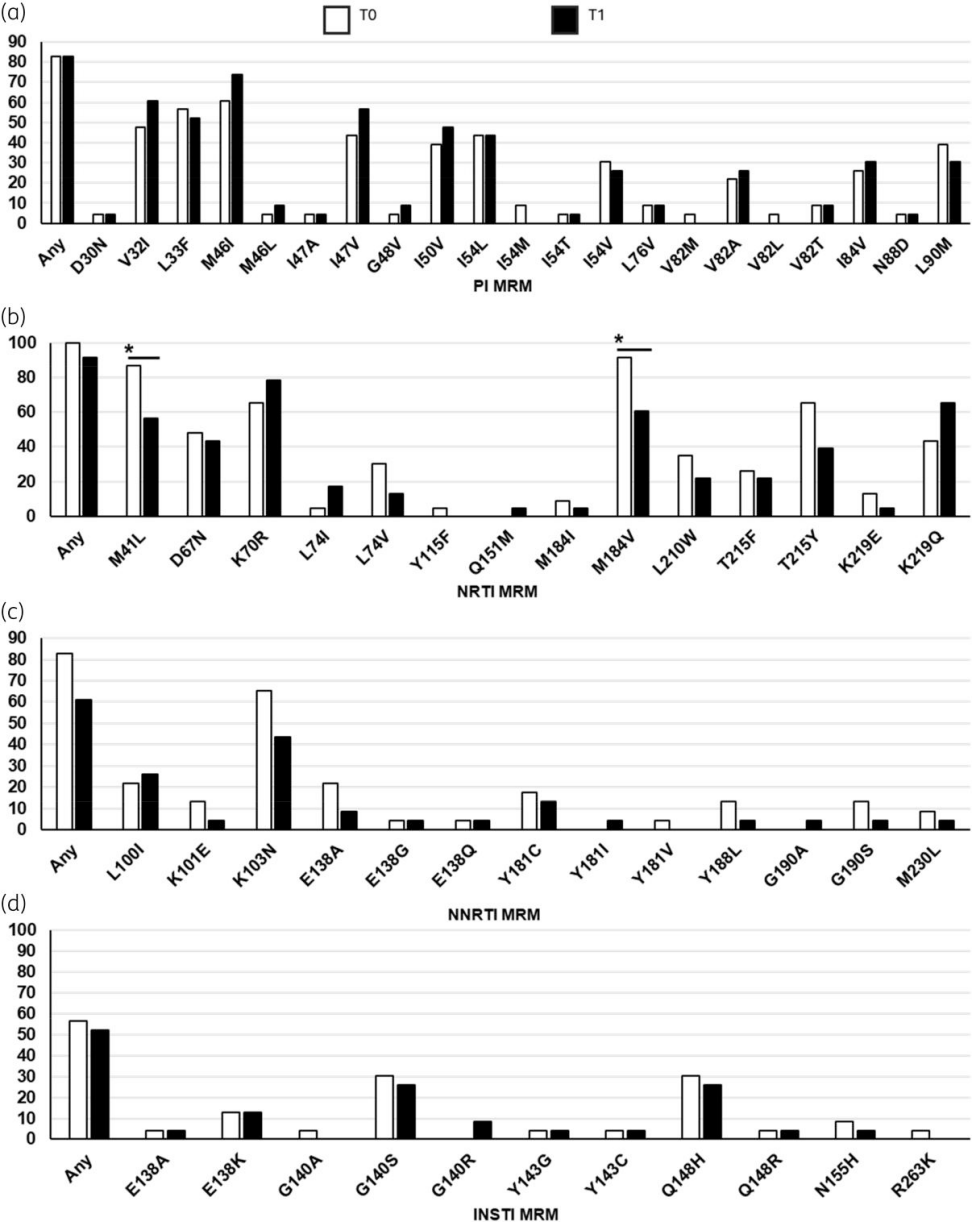
Overview of MRM prevalence detected through HIV-1 DNA NGS from longitudinal PBMC samples of HTE MDR PWH. Bar plots report prevalence of MRMs according to HIVdb ver 9.8 as detected through NGS (set at 5% cut-off) at T0 (white bars) and T1 (black bars) longitudinal samples. (a–d) MRM to PI, NRTI, NNRTI and INSTI, respectively. Only statistically significant differences (*P* value < 0.05, according to McNemar test for matched pairs) are indicated by an asterisk.

**Figure 2.**
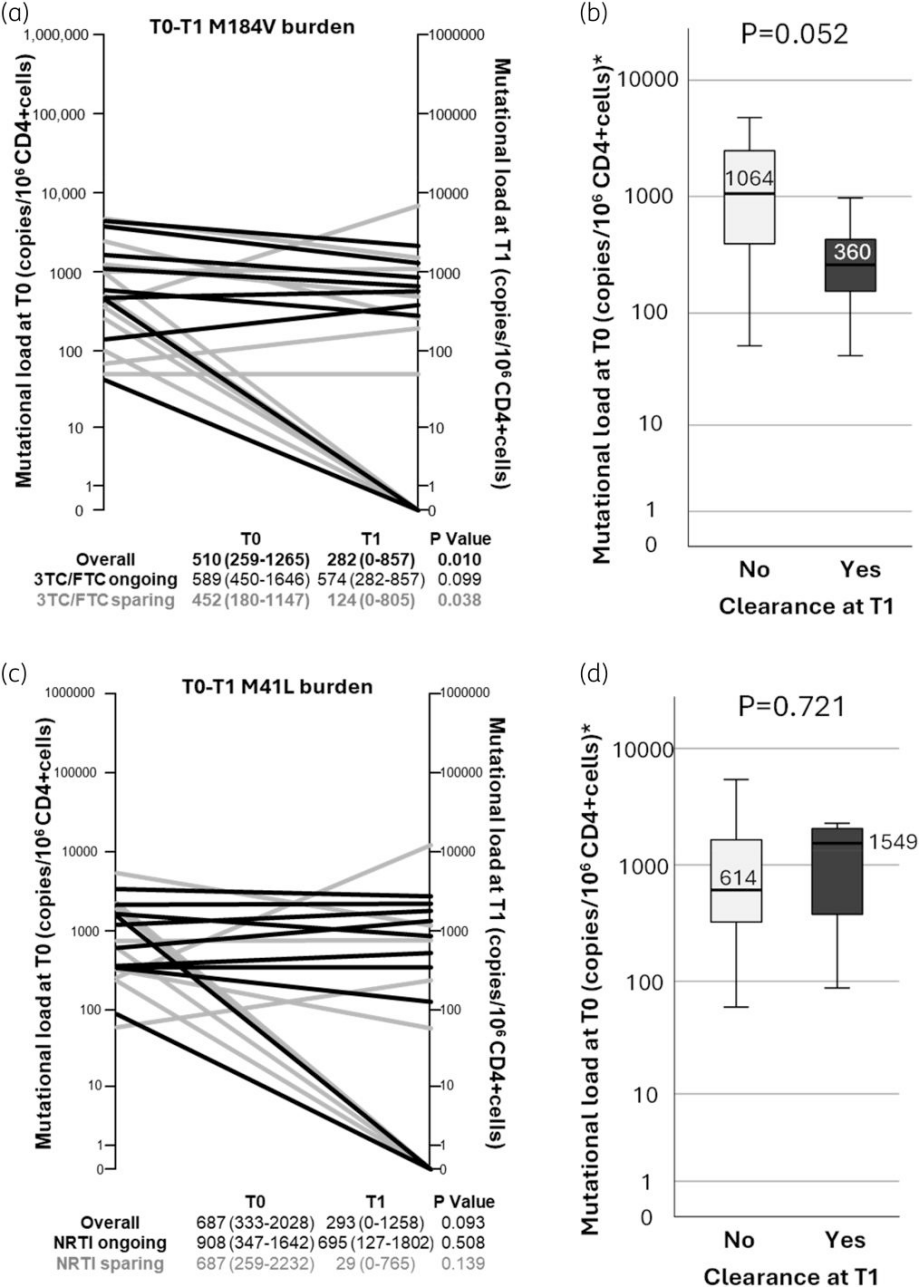
Burden of M184V and M41L detected through HIV-1 DNA NGS from longitudinal PBMC samples of HTE MDR PWH. (a) Spaghetti plot of M184V mutational load levels from T0 to T1. Black lines represent individuals under 3TC or FTC pressure; grey lines represent individuals not currently receiving 3TC or FTC. The table below indicates median (IQR) levels according to treatment. (b) Boxplot representing T0 mutational load levels according to clearance or maintenance of M184V at T1. (c) Spaghetti plot of M41L mutational load levels from T0 to T1. Black lines represent individuals under NRTI pressure; grey lines represent individuals not currently receiving NRTI. The table below indicates median (IQR) levels according to treatment. (d) Boxplot representing T0 mutational load levels according to clearance or maintenance of M41L at T1. *P* value according to Wilcoxon test for matched pairs.

Concerning specific MRM, the proportion of individuals harbouring RTI mutations decreased over time, but a significant decrease (T1–T0) was observed only for M184V (T0, 91.3%; T1, 60.9%; *P* = 0.016) and M41L (T0, 87.0; T1, 56.5%; *P* = 0.016) (Figure 1). No significant difference in the change of mutational load from T0 to T1 was found for any mutation with the exception of M184V, which significantly decreased over time (*P* = 0.010; Figure 2a). This decrease was observed regardless of 3TC/FTC pressure, even though it was more pronounced in individuals who were receiving a 3TC-/FTC-sparing salvage regimen.

Individuals who lost M184V (7 out 21, 33.3%) had a lower burden at T0 compared with those who had the mutation persistently detectable (Figure 2b). M184V was more likely to be cleared in individuals with T0 mutational load of <1000 versus of ≥1000 copies/10^6^ CD4+ cells at T0 (7 out of 13, 53.8% versus 0 out of 8, 0%; *P* = 0.018). The overall mutation burden of M41L decreased from T0 to T1 with a trend towards significance (*P* = 0.093), regardless of NRTI pressure (Figure 2c), and mutation clearance was not associated with T0 mutational load levels (Figure 2d). The detection percentage of M184V and M41L mutations and the corresponding amount of HIV DNA for each individual are reported in Tables S2–S3.

## Discussion

This study highlights the dynamics of HIV-1 resistance mutations in virologically suppressed HTE MDR individuals, one of the most vulnerable populations in the HIV field. Our longitudinal analysis showed that the overall prevalence of at least 3-class resistance and the number of MRM remained stable over 1 year. Specifically, resistance to PI and INSTI (and partly to NRTI) remained unchanged, as expected in individuals mostly receiving dolutegravir- and/or darunavir-based salvage regimens. By contrast, a significant decrease in NNRTI resistance was observed in individuals not receiving NNRTIs, while resistance levels remained stable in those on NNRTI therapy (especially etravirine). Due to the potential underestimation and fluctuation related to HIV DNA resistance testing, we cannot exclude that resistance reversal occurred before T0. However, this apparent resistance reversal is reasonable, considering that individuals off NNRTI therapy had stopped using the class about 8 years earlier. This finding is in line with a study showing 80% clearance of NNRTI resistance in HIV-1 DNA after 5 years of withdrawal.^6^ Moreover, it should be considered that at T0, 35% of historical NNRTI MRM detected through Sanger were not re-detected by NGS; thus, a further apparent clearance after one additional year is also reasonable.

Regarding NRTI mutations, despite overall stability, only M184V and M41L showed a significant decline. Specifically, M184V, associated with 3TC/FTC resistance, decreased from 91.3% at T0 to 60.9% at T1, with its disappearance significantly associated with a baseline mutational load of <1000 copies/10^6^ CD4+ cells. No such association was found for M41L.

Occasional NRTI mutation clearance, especially under NRTI-sparing regimens, has been already observed,^6,7^ but we did not observe that the absence of 3TC/FTC pressure contributed to the disappearance of M184V. This was likely due to the long and heterogeneous treatment history of the HTE individuals. We hypothesize that suppressive cART might dilute and, in some cases, clear the burden of M184V in the reservoir considering also the fitness cost of this mutation.^11^

Given the challenges on collecting longitudinal data on HTE MDR individuals with long-term virological suppression, our findings contribute to the understanding of resistance dynamics in these vulnerable individuals. However, this study has certain limitations. The short observation period (12 months) limits our ability to assess long-term disappearance of MRMs, especially PI- and INI-associated mutations, as 70% of the cohort remained on these drugs. Additionally, the small sample size (in a context of heterogeneous treatment history) may limit the generalizability of our findings for whom it was not possible to evaluate factors associated with resistance clearance with predictive models.

In conclusion, short-term waning of drug resistance mutations in HIV-1 DNA is uncommon in virologically suppressed HTE MDR individuals; however, the decline of M184V highlights the dynamic nature of the viral reservoir under virological suppression. These results underscore the importance of personalized treatment strategies and ongoing monitoring to optimize outcomes in this vulnerable population, where drug recycling or the evaluation of potential cross-resistance to novel drugs (such as doravirine and islatravir) within existing drug classes may be necessary. In this context, HIV-1 DNA resistance might be useful in orienting subsequent therapeutic decisions.

## Supplementary Material

dkaf349_Supplementary_Data

## Supplementary data

Tables S1–S3 are available as Supplementary data at *JAC* Online.
