# Supplementary material for: Evaluation of HIV-1 DNA resistance evolution in highly treatment-experienced and multi-resistant individuals under suppressive antiretroviral therapy: a longitudinal study from the PRESTIGIO Registry
Source: J Antimicrob Chemother. 2025 Sep 24;80(11):3101–6. doi: 10.1093/jac/dkaf349 (PMC12596042; doi:10.1093/jac/dkaf349)
Supplement: dkaf349_Supplementary_Data [file dkaf349_supplementary_data.zip › Table_S2_S3_08082025.docx]

| **Code^a^** | **M184V: T0** | | | **M184V: T1** | | | **Change T1- T0** | | |
| --- | --- | --- | --- | --- | --- | --- | --- | --- | --- |
|  | **Frequency**  **(%)** | **Mutational Load**  **(copies/10^6^CD4+)** | **HIV-DNA amount**  **(copies/10^6^CD4+)** | **Frequency**  **(%)** | **Mutational Load (copies/10^6^CD4+)** | **HIV-DNA amount**  **(copies/10^6^CD4+)** | **Frequency**  **(%)** | **Mutational Load (copies/10^6^CD4+)** | **HIV-DNA amount**  **(copies/10^6^CD4+)** |
| **104-0008** | 11.0 | 141 | 1281 | 23.3 | 384 | 1644 | 12.3 | 243 | 363 |
| 104-0015 | 37.1 | 4795 | 12917 | 66.1 | 1536 | 2322 | 29.0 | -3259 | -10595 |
| **104-0016** | 96.2 | 3767 | 3915 | 17.9 | 1298 | 7242 | -78.3 | -2469 | 3327 |
| 104-0021 | 47.1 | 1265 | 2687 | 13.1 | 496 | 3781 | -34.0 | -769 | 1094 |
| 104-0029 | 15.2 | 1030 | 6756 | 38.8 | 1114 | 2874 | 23.5 | 84 | -3882 |
| **104-0033** | 58.4 | 4403 | 7533 | 34.3 | 2137 | 6229 | -24.1 | -2266 | -1304 |
| **104-0038** | 28.3 | 450 | 1589 | 0.0 | 0 | 1986 | -28.3 | -450 | 397 |
| **104-0040** | 15.6 | 42 | 272 | 0.0 | 0 | 1154 | -15.6 | -42 | 882 |
| 107-0006 | 15.3 | 395 | 2588 | 53.1 | 6986 | 13162 | 37.8 | 6591 | 10574 |
| 107-0007 | 12.5 | 510 | 4067 | 0.0 | 0 | 5373 | -12.5 | -510 | 1306 |
| 111-0007 | 5.5 | 101 | 1835 | 0.0 | 0 | 1905 | -5.5 | -101 | 70 |
| **111-0008** | 31.3 | 1097 | 3501 | 20.9 | 667 | 3190 | -10.4 | -430 | -311 |
| **111-0010** | 30.0 | 476 | 1585 | 19.0 | 574 | 3027 | -11.1 | 98 | 1442 |
| **111-0013** | 28.4 | 589 | 2078 | 12.1 | 282 | 2327 | -16.2 | -307 | 249 |
| 120-0002 | 27.9 | 259 | 929 | 0.0 | 0 | 1840 | -27.9 | -259 | 911 |
| 125-0002 | 8.7 | 52 | 596 | 29.7 | 51 | 173 | 21.0 | -1 | -423 |
| **125-0003** | 99.5 | 1646 | 1655 | 99.0 | 857 | 866 | -0.5 | -789 | -789 |
| 239-0003 | 15.6 | 980 | 6264 | 0.0 | 0 | 7333 | -15.6 | -980 | 1069 |
| 239-0005 | 9.5 | 70 | 744 | 13.0 | 196 | 1508 | 3.6 | 126 | 764 |
| 242-0003 | 43.9 | 2481 | 5652 | 20.1 | 265 | 1323 | -23.8 | -2216 | -4329 |
| 242-0006 | 7.0 | 360 | 5122 | 0.0 | 0 | 2746 | -7.0 | -360 | -2376 |
| **Median (IQR)** | 15.6 (11.8;34.2) | 510  (200;1181) | 2078  (1433;3991) | 13.1  (0.0;26.5) | 265  (0.0;890) | 2327  (1742;4577) | -12.5  (-24.0;7.9) | -360  (-874;-21) | 397  (-367;1081) |
| **P-Value** |  |  |  |  |  |  | 0.182 | **0.010** | 0.258 |

**Table S2. T0 and T1 frequency, mutational load and HIV-DNA levels of individuals harbouring M184V**

a. in bold individuals receiving 3TC or FTC. P-value calculated using the Wilcoxon signed-rank test.

**Table S3. T0 and T1 frequency, mutational load and HIV-DNA levels of individuals harbouring M41L.**

| **Code^a^** | **M41L: T0** | | | **M41L: T1** | | | **Change T1- T0** | | |
| --- | --- | --- | --- | --- | --- | --- | --- | --- | --- |
|  | **Frequency**  **(%)** | **Mutational Load**  **(copies/10^6^CD4+)** | **HIV-DNA amount**  **(copies/10^6^CD4+)** | **Frequency**  **(%)** | **Mutational Load (copies/10^6^CD4+)** | **HIV-DNA amount**  **(copies/10^6^CD4+)** | **Frequency**  **(%)** | **Mutational Load (copies/10^6^CD4+)** | **HIV-DNA amount**  **(copies/10^6^CD4+)** |
| **104-0008** | 27.1 | 347 | 1281 | 21.2 | 348 | 1644 | -5.9 | 1 | 363 |
| 104-0015 | 42.0 | 5424 | 12917 | 50.4 | 1171 | 2322 | 8.4 | -4253 | -10595 |
| **104-0016** | 30.7 | 1203 | 3915 | 24.9 | 1802 | 7242 | -5.8 | 599 | 3327 |
| 104-0021 | 85.2 | 2290 | 2687 | 0.0 | 0 | 3781 | -85.2 | -2290 | 1094 |
| 104-0029 | 9.1 | 616 | 6756 | 3.7 | 106 | 2874 | -5.4 | -510 | -3882 |
| **104-0033** | 45.1 | 3397 | 7533 | 44.0 | 2743 | 6229 | -1.0 | -654 | -1304 |
| **104-0038** | 21.5 | 342 | 1589 | 6.4 | 127 | 1986 | -15.1 | -215 | 397 |
| **104-0040** | 32.4 | 88 | 272 | 0.0 | 0 | 1154 | -32.4 | -88 | 882 |
| 107-0006 | 10.0 | 259 | 2588 | 93.5 | 12310 | 13162 | 83.5 | 12051 | 10574 |
| 107-0007 | 54.9 | 2232 | 4067 | 3.3 | 179 | 5373 | -51.6 | -2053 | 1306 |
| 111-0007 | 41.3 | 758 | 1835 | 40.2 | 766 | 1905 | -1.1 | 8 | 70 |
| **111-0008** | 61.3 | 2146 | 3501 | 69.5 | 2217 | 3190 | 8.2 | 71 | -311 |
| 111-0009 | 6.4 | 324 | 5040 | 6.5 | 58 | 903 | 0.1 | -266 | -4137 |
| **111-0010** | 22.9 | 363 | 1585 | 17.4 | 528 | 3027 | -5.5 | 165 | 1442 |
| **111-0013** | 29.5 | 614 | 2078 | 57.8 | 1344 | 2327 | 28.2 | 730 | 249 |
| 120-0002 | 25.2 | 234 | 929 | 0.0 | 0 | 1840 | -25.2 | -234 | 911 |
| **125-0003** | 99.2 | 1642 | 1655 | 99.6 | 862 | 866 | 0.3 | -780 | -789 |
| 239-0003 | 24.7 | 1548 | 6264 | 1.1 | 78 | 7333 | -23.6 | -1470 | 1069 |
| 239-0005 | 8.1 | 60 | 744 | 15.8 | 239 | 1508 | 7.8 | 179 | 764 |
| 242-0006 | 37.3 | 1909 | 5122 | 2.5 | 68 | 2746 | -34.8 | -1841 | -2376 |
| **Median (IQR)** | 30.1  (22.2;43.6) | 687  (333;2027) | 2637  (1587; 5081) | 16.6  (2.9;47.2) | 293  (0;1258) | 2536.  (1742;4577) | -5.4  (-24.4;4.0) | -224  (-1125; 118) | 379  (-1046; 1081) |
| **P-value** |  |  |  |  |  |  | 0.165 | 0.091 | 0.648 |

a. in bold individuals were receiving NRTI. P-value calculated using the Wilcoxon signed-rank test.
