# Supplementary material for: Evaluation of HIV-1 DNA resistance evolution in highly treatment-experienced and multi-resistant individuals under suppressive antiretroviral therapy: a longitudinal study from the PRESTIGIO Registry
Source: J Antimicrob Chemother. 2025 Sep 24;80(11):3101–6. doi: 10.1093/jac/dkaf349 (PMC12596042; doi:10.1093/jac/dkaf349)
Supplement: dkaf349_Supplementary_Data [file dkaf349_supplementary_data.zip › TableS1_08082025.docx]

| **MRM detected with NGS-GRT set at 5%, median (IQR)** | **T0** | **T1** | **P Value^a^** |
| --- | --- | --- | --- |
| **Any MRM** | 12 (10-16) | 13 (8-14) | 0.384 |
| **PI** | 6 (2-7) | 6 (2-6) | 0.617 |
| *Ongoing in regimen* | 4 (2-6) | 6 (3-7) | 0.174 |
| *Sparing in regimen* | 7 (6-9) | 6 (3-7) | 0.109 |
| **NRTI** | 6 (4-6) | 4 (2-7) | 0.170 |
| *Ongoing in regimen* | 5 (4-6) | 4 (3-6) | 0.317 |
| *Sparing in regimen* | 6 (4-6) | 5 (2-7) | 0.303 |
| *Any TAM overall* | 4 (3-5) | 3 (2-5) | 0.274 |
| *Any TAM NRTI ongoing in regimen* | 4 (3-5) | 3 (2-5) | 0.336 |
| *Any TAM NRTI sparing in regimen* | 4 (3-5) | 4 (2-5) | 0.472 |
| **NNRTI** | 2 (1-3) | 1 (0-2) | 0.153 |
| *Ongoing in regimen* | 2 (2-3) | 2 (1-3) | 0.832 |
| *Sparing in regimen* | **2 (1-3)** | **0 (0-1)** | **0.027** |
| **INSTI^b^** | 1 (0-1) | 1 (0-1) | 0.822 |
| ^a^Wilcoxon test for matched pairs; ^b^Only one person was receiving an INSTI sparing regimen thus the median number of INSTI MRM was calculated in the overall population. | | | |

**Table S1. Number of major resistance mutations (MRM) in longitudinal PBMC samples of virologically suppressed HTE-MDR PWH according to specific drug class pressure**
